# Identification and quantification of mepirapim and acetyl fentanyl in authentic human whole blood and urine samples by GC–MS/MS and LC–MS/MS

**DOI:** 10.1007/s11419-017-0384-7

**Published:** 2017-09-12

**Authors:** Akira Mochizuki, Hiroko Nakazawa, Noboru Adachi, Kenichi Takekawa, Hideki Shojo

**Affiliations:** 1 Forensic Science Laboratory, Yamanashi Prefectural Police Headquarters, 312-4 Kubonakajima, Isawa, Fuefuki, Yamanashi 406-0036 Japan; 2 Department of Legal Medicine, Graduate Faculty of Interdisciplinary Research, University of Yamanashi, 1110 Shimokato, Chuo, Yamanashi 409-3898 Japan

**Keywords:** Mepirapim, Synthetic cannabinoid, Acetyl fentanyl, GC–MS/MS, LC–MS/MS, Whole blood and urine

## Abstract

**Purpose:**

We encountered a curious case in which two male subjects self-administered mepirapim plus acetyl fentanyl by different routes, i.e., intravenously and by inhalation. We have thus established a detailed procedure for quantification of mepirapim and acetyl fentanyl in whole blood and urine specimens using gas chromatography (GC)–tandem mass spectrometry (MS/MS).

**Methods:**

The GC–MS/MS method was validated for linearity, extraction recovery, accuracy, and precision. Liquid chromatography–MS/MS was also used for identification of the target compounds.

**Results:**

Good linearity and reproducibility were achieved in the range of 20–1000 ng/g for both target compounds in both matrices. The concentrations of mepirapim in heart whole blood, femoral vein whole blood, and urine of the deceased individual with administration by intravenous injection were 593, 567, and 527 ng/g, respectively; those of acetyl fentanyl were 155, 125, and 126 ng/g, respectively. The mepirapim and acetyl fentanyl concentrations in the urine specimen of the surviving individual who had administered them by inhalation were 4900 and 570 ng/g, respectively.

**Conclusions:**

To our knowledge, with the exception of a brief mention of a mepirapim concentration in a serum sample in emergency medicine, there are no reported data on the identification and quantification of mepirapim in biological samples. Mepirapim is a new synthetic cannabinoid. The concentration profiles of unchanged mepirapim in whole blood and urine were quite different and unique. A detailed clarification of such uniqueness is under way in our laboratory.

## Introduction

**Fig. 1:**
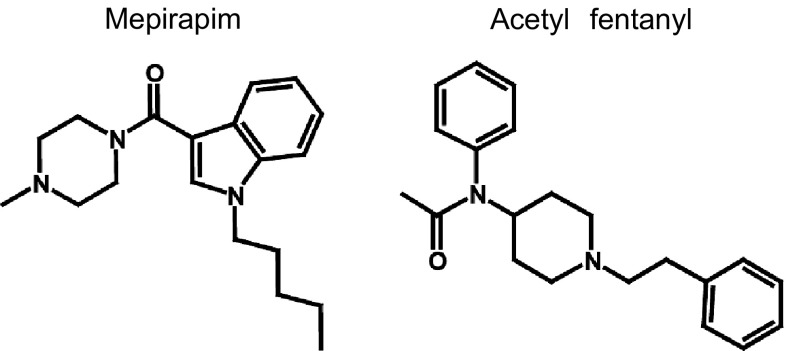
Structures of mepirapim and acetyl fentanyl

Illicit psychoactive substances (e.g., synthetic cannabinoids, cathinone derivatives, and synthetic opioids) have become a serious threat worldwide as designer drugs of abuse [1–3]. Mepirapim is a new and unique synthetic cannabinoid that was first identified in herbal blends in Japan [4]. This compound differs from JWH-018 in that it has a 4-methylpiperazine group in place of the naphthyl group (Fig. 1) [5]. Similar to JWH-018, mepirapim is thought to be a potent synthetic cannabinoid that is avidly bound to both the central CB_1_ and the peripheral CB_2_ receptors [6]. However, the detailed pharmacological and toxicological properties of mepirapim are unknown.

Acetyl fentanyl is a synthetic fentanyl analogue in which the propionyl group of fentanyl is replaced by an acetyl group (Fig. 1) [7]. Acetyl fentanyl has recently been encountered in several clinical and forensic case studies [8–15]. In the United States, 14 overdose deaths were reported in Rhode Island from March through May of 2013 [9], and in Japan, acetyl fentanyl was identified in illegal products in 2014 [1]. Acetyl fentanyl acts as a µ-opioid receptor agonist and is associated with euphoria, drowsiness, and respiratory depression [3]. Of concern is that the range between the effective and lethal doses is narrower than that of morphine [3].

In forensic toxicology, investigation of the concentrations in biological fluid samples is critical for estimating the cause of death and the time elapsed after dosing. Here, we encountered two individuals, one of a surviving individual and the other one of fatal poisoning, both of which involved a white powder product called “Angela” administered via different routes. In this study, we identified and quantified mepirapim and acetyl fentanyl from postmortem specimens in the fatal case using gas chromatography–tandem mass spectrometry (GC–MS/MS) and liquid chromatography–tandem mass spectrometry (LC–MS/MS). In the surviving individual, we analyzed the mepirapim and acetyl fentanyl concentrations in a urine specimen.

## Case history

In December 2013, a case of drug abuse involving two individuals occurred, one fatal and the other non-fatal. According to the statement taken from the surviving individual (male, aged 40s), both he and the deceased (male, aged 60s) self-administered a white powder product called “Angela.” The surviving individual used the product by inhalation, whereas the deceased self-administered the product (approximately 50–60 mg) via intravenous injection. Approximately 10 h after dosing, the surviving subject recognized that the other subject had died.

For the victim of fatal poisoning, an autopsy was performed, and postmortem biological fluid samples (heart whole blood, femoral vein whole blood, and urine) were collected. For the surviving individual, a urine sample was collected approximately 16 h after inhalation. The “Angela” product found at the scene was seized by the authorities. Drug analyses for both subjects were performed at our department at the request of judicial authorities.

## Materials and methods

### Materials

Mepirapim hydrochloride, acetyl fentanyl, and acetyl fentanyl-*d*_5_ were purchased from Cayman Chemical (Ann Arbor, MI, USA). Other common chemicals used in this study were of the highest purity commercially available.

To prepare the calibrators and quality controls, we collected human whole blood and urine from two healthy volunteers with no known diseases and no history of drug use. Informed consent was obtained from both participants who supplied blood and urine for use as blank matrices. The samples obtained in December 2013 were stored at −80 °C until analysis.

### Extraction procedure

An aliquot of 0.2 g of blood or urine was placed in a polypropylene vial prepared with 100 µL of acetyl fentanyl-*d*_5_ internal standard (0.2 µg/mL), 1 mL of saturated NaCl, and 1 mL of 1% Na_2_CO_3_. Liquid–liquid extraction was performed with 4 mL of diethyl ether by vortexing for 2 min. After centrifugation (4000 rpm for 5 min), the organic layer was transferred to another vial and evaporated; the residue was reconstituted in 100 µL of ethyl acetate (for GC–MS/MS) or methanol (for LC–MS/MS).

### GC–MS and GC–MS/MS conditions

Identification of mepirapim and acetyl fentanyl was performed by full scan GC–MS analysis. Quantitative measurements were carried out by multiple reaction monitoring (MRM) analysis of GC–MS/MS. Briefly, the GC–MS and GC–MS/MS instrument was a Bruker 456-GC gas chromatograph connected to a SCION TQ mass spectrometer (Bruker Daltonics, Billerica, MA, USA). GC conditions were as follows: separation column, Rtx-5Sil MS fused-silica capillary (30 m × 0.25 mm i.d., 0.25 µm film thickness; Restek, Bellefonte, PA, USA); injector temperature, 250 °C; interface temperature, 280 °C; injection mode, splitless; injection volume, 1 µL; carrier gas, He 1.0 mL/min; oven temperature program, initial temperature at 100 °C followed by ramp at 20 °C/min up to 300 °C (10 min hold). MS conditions included the following: ion source temperature, 220 °C; ionization mode, electron ionization (EI) at 70 eV; emission current, 80 µA; scan mode, full scan (for qualification, scan range; *m*/*z* 45–500) or MRM (for quantifier and qualifiers, ion transitions: Table 1).

**Table 1:** Ion transitions and collision energies for mepirapim, acetyl fentanyl, and acetyl fentanyl-*d*_5_ (internal standard, IS) used for gas chromatography–tandem mass spectrometry (GC–MS/MS) analysis

Analyte	Ion transition	Collision energy (V)
Mepirapim	313 > 214^a^ 230 > 173 214 > 144	15 15 20
Acetyl fentanyl	231 > 146^a^ 231 > 158 188 > 130	20 15 35
Acetyl fentanyl-*d*_5_ (IS)	236 > 151^a^ 236 > 163	20 15

^a^ The transition ions used for quantification

### LC–MS/MS conditions

Identification of mepirapim and acetyl fentanyl was also performed by MS/MS and MS^3^ (multistage) product scan analysis by LC–MS^*n*^. Briefly, LC–MS/MS was performed on a Prominence LC system (Shimadzu, Kyoto, Japan) connected to an LTQ XL ion trap mass spectrometer (Thermo Fisher Scientific, Waltham, MA, USA). LC conditions were as follows: separation column, XBridge C18 column (150 × 2.1 mm i.d., particle size 5 µm, Waters Corp., Milford, MA, USA); injection volume, 2 µL; flow rate, 0.2 mL/min; elution mode, isocratic with 10 mM ammonium acetate in water/methanol (35:65, v/v); column temperature, 40 °C. MS conditions were as follows: interface, electrospray ionization (ESI) mode; polarity, positive; capillary temperature, 250 °C; capillary voltage, 25 V; normalized collision energy, 35%; scan mode, product scan; precursor ions, *m*/*z* 314 (MS/MS) and 314 > 214 (MS^3^) for mepirapim, *m*/*z* 323 (MS/MS) and 323 > 188 (MS^3^) for acetyl fentanyl.

### Validations

Calibration curves were prepared with drug-free whole blood or urine fortified with mepirapim and acetyl fentanyl standard solutions at 20, 50, 100, 200, 500, and 1000 ng/g. Extraction recovery, accuracy, and precision were obtained for both compounds in both whole blood and urine.

## Results

### GC–MS and LC–MS/MS spectra of mepirapim and acetyl fentanyl

**Fig. 2:**
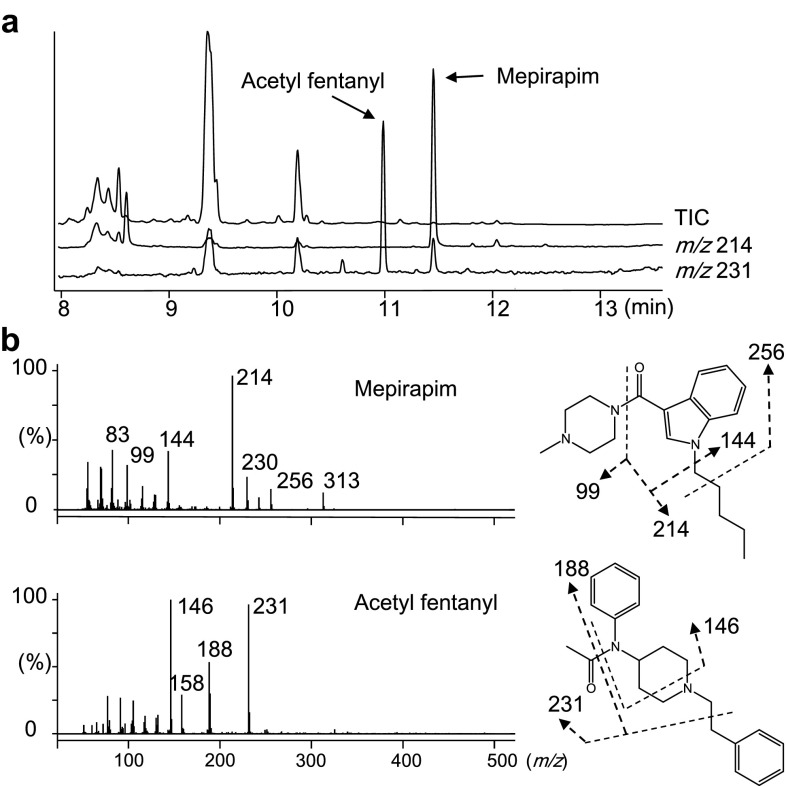
Gas chromatography–mass spectrometry analysis of mepirapim and acetyl fentanyl in femoral vein whole blood. Total ion current chromatogram (TIC) and extracted ion chromatograms at *m*/*z* 214 and 231 (**a**). Electron ionization mass spectra of mepirapim and acetyl fentanyl (**b**)

**Fig. 3:**
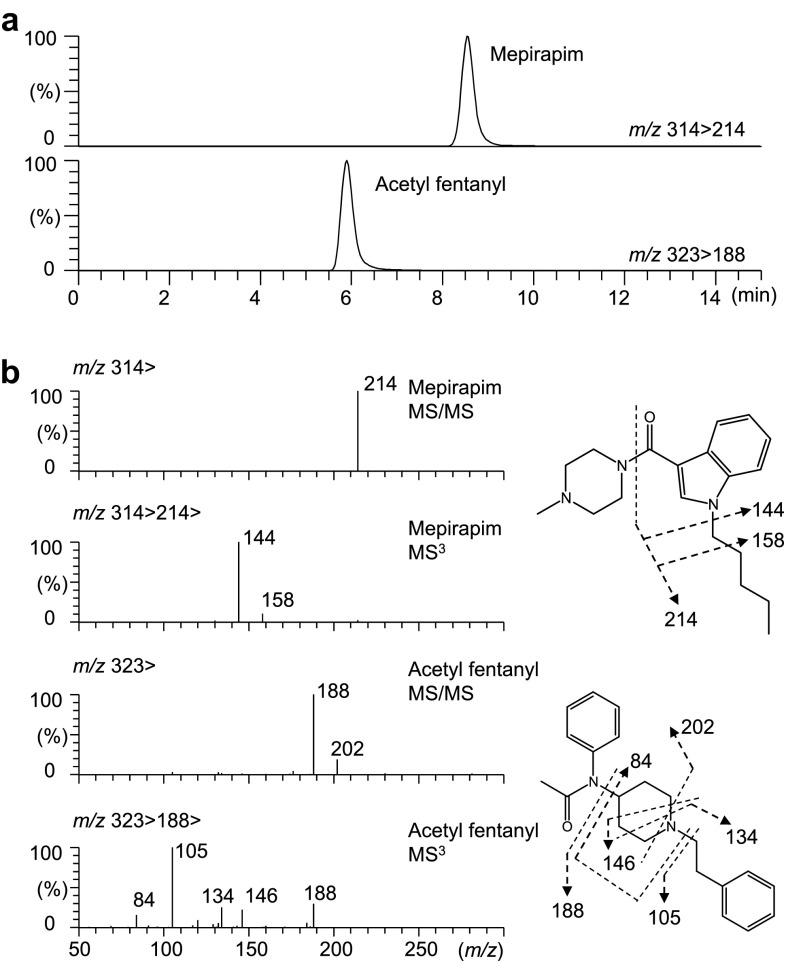
Liquid chromatography–tandem mass spectrometry analysis of mepirapim and acetyl fentanyl in femoral vein whole blood. Multiple reaction monitoring chromatograms for mepirapim and acetyl fentanyl (**a**). Electrospray ionization product ion spectra of mepirapim and acetyl fentanyl (**b**)

Qualitative analyses of the biological fluid samples were carried out under the GC–MS and LC–MS/MS conditions described above. Figures 2 and 3 show the data from femoral vein whole blood samples by GC–MS and LC–MS/MS, respectively. The mass spectra and retention times for the peaks that appeared in the chromatograms coincided with those of the reference standard samples.

### Validation of the quantitative method by GC–MS/MS

The extraction recovery of mepirapim and acetyl fentanyl was evaluated using drug-free blood or urine fortified with 50 and 200 ng/g for both mepirapim and acetyl fentanyl. The recovery of mepirapim and acetyl fentanyl in whole blood was not less than 81.0 and 88.3%, respectively (Table 2; *n* = 5 each). In addition, the recovery of mepirapim and acetyl fentanyl in urine was not less than 87.2 and 88.9%, respectively (*n* = 5 each).

**Table 2:** Extraction recoveries of mepirapim and acetyl fentanyl

Concentration added (ng/g)	Recovery (%)			
	Whole blood		Urine	
	Mepirapim	Acetyl fentanyl	Mepirapim	Acetyl fentanyl
50	81.0 ± 10.1	88.3 ± 4.0	87.3 ± 10.2	88.9 ± 5.2
200	84.8 ± 8.3	91.0 ± 3.5	87.2 ± 8.7	93.1 ± 6.3

The values are shown as mean ± standard deviation (SD) (*n* = 5)

Quantitative analyses of mepirapim and acetyl fentanyl in whole blood by GC–MS/MS showed high linearity from 20 to 1000 ng/g (*y* = 0.001891*x* − 0.01486, *r* ^*2*^ = 0.9971 and *y* = 0.01165*x* − 0.01201, *r* ^*2*^ = 0.9999, respectively; *n* = 5 each). The equivalent analyses of both compounds in urine also showed linearity in the same range (*y* = 0.002009*x* – 0.01616, *r* ^*2*^ = 0.9955 and *y* = 0.01310*x* – 0.06607, *r* ^*2*^ = 0.9985, respectively; *n* = 5 each). The detection limits of both mepirapim and acetyl fentanyl were 0.1–1.0 ng/g. The quantification limits of both compounds were 10–20 ng/g.

For mepirapim, the intraday accuracy bias was not greater than 14.2%, and the interday accuracy bias not greater than 14.6%, at all calibrator concentrations for both matrices. For acetyl fentanyl, the intraday accuracy bias was not greater than 5.8%, and the interday accuracy bias not greater than 8.6%, through the range of calibrator concentrations for both matrices (Table 3).

The intraday precision for mepirapim was not greater than 14.2%, and the interday precision was not greater than 14.7%, through the range of calibrator concentrations for both matrices. The intraday precision for acetyl fentanyl was not greater than 9.1%, and the interday precision was not greater than 5.4%, through the range of calibrator concentrations for both matrices.

The above validation data show that the GC–MS/MS quantification method proposed for mepirapim and acetyl fentanyl in human whole blood and urine is generally robust.

### A white powder product called “Angela”

The white powder product called “Angela” was analyzed; the powder was composed of 73.2 ± 0.4% mepirapim and 18.9 ± 0.2% acetyl fentanyl (w/w) (*n* = 3 each).

**Table 3:** Intraday and interday precision and accuracy data for the determination of mepirapim and acetyl fentanyl at various concentrations in whole blood and urine by GC–MS/MS

Concentration (ng/g)	Mepirapim				Acetyl fentanyl			
	Intraday		Interday		Intraday		Interday	
	Accuracy bias (%)	Precision (%)	Accuracy bias (%)	Precision (%)	Accuracy bias (%)	Precision (%)	Accuracy bias (%)	Precision (%)
Whole blood								
20	+5.7	9.8	+5.7	7.5	+1.8	1.5	+1.1	1.7
50	−8.8	7.7	−8.8	11.8	−3.2	1.0	−1.3	2.9
100	−12.1	7.9	−12.1	7.7	−3.0	1.1	−8.6	2.9
200	−2.3	6.1	−2.3	9.1	0.0	0.5	−1.2	1.9
500	+6.1	6.5	+6.1	2.7	+2.4	1.7	+2.5	1.6
1000	+11.4	8.0	+11.4	3.5	+2.1	0.7	+6.5	3.4
Urine								
20	+3.7	14.2	+13.2	11.5	+0.5	9.1	+4.0	1.5
50	−14.2	13.9	−1.0	14.7	−5.8	3.8	−2.0	3.1
100	−8.2	13.8	−13.5	12.4	−1.5	5.8	−6.7	5.0
200	+3.1	7.7	−5.8	10.2	+0.6	4.6	+2.0	5.4
500	+3.3	11.3	−1.0	9.6	+3.0	1.8	−0.8	2.7
1000	+9.3	6.2	+14.6	5.7	+3.6	3.3	+6.7	4.6

Each value is the mean of five determinations

### Blood and urine concentrations of mepirapim and acetyl fentanyl in the deceased individual

As shown in Table 4, the concentrations of mepirapim in heart whole blood, femoral vein whole blood, and urine were 593, 567, and 527 ng/g, respectively. For acetyl fentanyl, concentrations in heart whole blood, femoral vein whole blood, and urine were 155, 125, and 126 ng/g, respectively. Results of the experiments were confirmed by quadruplicate assays.

**Table 4:** Concentrations of mepirapim and acetyl fentanyl in human whole blood and urine

Sample	Mepirapim (ng/g)	Acetyl fentanyl (ng/g)
Deceased individual		
Heart whole blood	593 ± 15	155 ± 1
Femoral vein whole blood	567 ± 18	125 ± 1
Urine	527 ± 45	126 ± 1
Surviving individual		
Urine	4900^a^	570^a^

Quantitative analyses of mepirapim and acetyl fentanyl in biological fluid samples were carried out by multiple reaction monitoring analysis of GC–MS/MS. The values are shown as mean ± SD (*n* = 4)

^a^ The urine concentrations in the surviving individual were estimated by GC–MS

### Urine concentrations of mepirapim and acetyl fentanyl in the surviving individual

From the quantitative analysis by GC–MS/MS, urine concentrations of mepirapim and acetyl fentanyl in the surviving individual were 4900 and 570 ng/g, respectively.

## Discussion

We encountered a case of substance abuse involving two individuals, one fatal and the other non-fatal, which involved the same product, “Angela,” self-administered intravenously and by inhalation, respectively. Although mepirapim was first identified in herbal blends in Japan [4], with the exception of a brief mention of serum mepirapim concentration in one of six poisoning cases encountered in emergency medicine [16], this is the first report dealing with identification and quantification of mepirapim in biological specimens. Acetyl fentanyl has also been identified repeatedly in Japan in herbal products sold through the Internet and/or quantified in human specimens [8–15]. The most common route of administration for acetyl fentanyl is intravenous injection [3, 11, 12]. In the present case, “Angela” was a white powder product rather than an herbal blend. For the surviving individual after inhalation, urine concentrations of mepirapim (4900 ng/g) and acetyl fentanyl (570 ng/g) were approximately ninefold and fivefold higher than those in the urine of the deceased individual with intravenous exposure, respectively. These results likely indicate that in the latter, death occurred before sufficient excretion and metabolism of these drugs, suggesting that the intravenous injection of both drugs was the cause of death by acute poisoning. Based on the statement from the surviving subject, it was estimated that the deceased self-administered approximately 30–40 mg mepirapim and 9–12 mg acetyl fentanyl via intravenous injection.

The present concentrations of mepirapim in blood samples (567–593 ng/g) were much higher than those of various other synthetic cannabinoids (0.1–199 ng/mL) in previous reports of poisoning deaths [2, 17–19]. Based on reports of acetyl fentanyl concentrations in biological fluid samples [3, 10–15], the concentrations of acetyl fentanyl in femoral vein and heart whole blood in the present case were relatively similar to those in previous reports, with acetyl fentanyl blood levels in fatal cases of 6–600 ng/mL [10], 250–260 ng/mL [11], 153 ng/mL [12], 270 ng/mL [13], 192–285 ng/mL [14], and 235 ng/mL [15].

The urinary levels of unchanged mepirapim in the deceased and surviving individuals were as high as 527 and 4900 ng/g, respectively (Table 4). Such high concentrations have never been encountered for other synthetic cannabinoids; the levels of unchanged synthetic cannabinoids in human urine specimens are generally very low, sub-nanogram per milliliter, and are frequently undetectable by conventional LC–MS/MS [20]. Therefore, the urinary excretion, pharmacokinetics, and metabolism of mepirapim may be quite different from those of other synthetic cannabinoids, which remains to be explored.

In the present report, urine concentrations of acetyl fentanyl were 126 and 570 ng/g for subjects in the fatal and non-fatal cases, respectively (Table 4). Previous reports have found urinary levels of acetyl fentanyl of 2600 ng/mL [11], 240 ng/mL [12], 3420 ng/mL [14], and 234 ng/mL [15], which can be easily identified and quantified by MS analysis. Urine specimens are the preferred method for testing of acetyl fentanyl because of the noninvasive nature and the ability to collect sufficient amounts.

In consideration of the above results, the cause of death for the deceased individual can be concluded to be synergistic acute poisoning by mepirapim and acetyl fentanyl.

## Conclusions

We encountered a curious case in which two male subjects self-administered mepirapim plus acetyl fentanyl by different routes, i.e., intravenously and by inhalation. We thus established a detailed procedure for quantification of mepirapim and acetyl fentanyl in whole blood and urine specimens by GC–MS/MS, also providing validation data. To our knowledge, this is the first report dealing with robust identification and quantification of mepirapim in whole blood and urine specimens from drug abusers. The levels of mepirapim in whole blood and urine were much higher than expected, which requires further investigation. This line of experiments is now in progress in our laboratory.

## References

[1] Uchiyama N, Shimokawa Y, Kawamura M, Kikura-Hanajiri R, Hakamatsuka T (2014). Chemical analysis of a benzofuran derivative, 2-(2-ethylaminopropyl)benzofuran (2-EAPB), eight synthetic cannabinoids, five cathinone derivatives, and five other designer drugs newly detected in illegal products. Forensic Toxicol.

[2] Gurney SMR, Scott KS, Kacinko SL, Presley BC, Logan BK (2014). Pharmacology, toxicology, and adverse effects of synthetic cannabinoid drugs. Forensic Sci Rev.

[3] Katselou M, Papoutsis I, Nikolaou P, Spiliopoulou C, Athanaselis S (2016). Old opioids, new concerns: the case of acetyl fentanyl. Forensic Toxicol.

[4] Uchiyama N, Shimokawa Y, Matsuda S, Kawamura M, Kikura-Hanajiri R, Goda Y (2014). Two new synthetic cannabinoids, AM-2201 benzimidazole analog (FUBIMINA) and (4-methylpiperazin-1-yl)(1-pentyl-1*H*-indol-3-yl)methanone (MEPIRAPIM), and three phenethylamine derivatives, 25H-NBOMe 3,4,5-trimethoxybenzyl analog, 25B-NBOMe, and 2C-N-NBOMe, identified in illegal products. Forensic Toxicol.

[5] Cayman chemical (2017) https://www.caymanchem.com/product/15388. Accessed June 2017

[6] Aung MM, Griffin G, Huffman JW, Wu M, Keel C, Yang B, Showalter VM, Abood ME, Martin BR (2000). Influence of the N-1 alkyl chain length of cannabimimetic indoles upon CB_1_ and CB_2_ receptor binding. Drug Alcohol Depend.

[7] Higashikawa Y, Suzuki S (2008). Studies on 1-(2-phenethyl)-4-(N-propionylanilino) piperidine (fentanyl) and its related compounds. VI. Structure–analgesic activity relationship for fentanyl, methyl-substituted fentanyls and other analogues. Forensic Toxicol.

[8] Stogner JM (2014). The potential threat of acetyl fentanyl: legal issues, contaminated heroin, and acetyl fentanyl “disguised” as other opioids. Ann Emerg Med.

[9] Lozier MJ, Boyd M, Stanley C, Ogilvie L, King E, Martin C, Lewis L (2015). Acetyl fentanyl, a novel fentanyl analog, causes 14 overdose deaths in Rhode Island, March-May 2013. J Med Toxicol.

[10] Poklis J, Poklis A, Wolf C, Mainland M, Hair L, Devers K, Chrostowski L, Arbefeville E, Merves M, Pearson J (2015). Postmortem tissue distribution of acetyl fentanyl, fentanyl and their respective nor-metabolites analyzed by ultrahigh performance liquid chromatography with tandem mass spectrometry. Forensic Sci Int.

[11] McIntyre IM, Trochta A, Gary RD, Malamatos M, Lucas JR (2015). An acute acetyl fentanyl fatality: a case report with postmortem concentrations. J Anal Toxicol.

[12] Yonemitsu K, Sasao A, Mishima S, Ohtsu Y, Nishitani Y (2016). A fatal poisoning case by intravenous injection of “bath salts” containing acetyl fentanyl and 4-methoxy PV8. Forensic Sci Int.

[13] Takase I, Koizumi T, Fujimoto I, Yanai A, Fujimiya T (2016). An autopsy case of acetyl fentanyl intoxication caused by insufflation of ‘designer drugs’. Leg Med.

[14] Fort C, Curtis B, Nichols C, Niblo C (2016). Acetyl fentanyl toxicity: two case report. J Anal Toxicol.

[15] Cunningham SM, Haikal NA, Kraner JC (2016). Fatal intoxication with acetyl fentanyl. J Forensic Sci.

[16] Fujita Y, Koeda A, Fujino Y, Onodera M, Kikuchi S, Niitsu H, Iwasaki Y, Usui K, Inoue Y (2016). Clinical and toxicological findings of acute intoxication with synthetic cannabinoids and cathinones. Acute Med Surg.

[17] Shanks KG, Dahn T, Terrell AR (2012). Detection of JWH-018 and JWH-073 by UPLC–MS–MS in postmortem whole blood casework. J Anal Toxicol.

[18] Behonick G, Shanks KG, Firchau DJ, Mathur G, Lynch CF, Nashelsky M, Jaskierny DJ, Meroueh C (2014). Four postmortem case reports with quantitative detection of the synthetic cannabinoid, 5F-PB-22. J Anal Toxicol.

[19] Hermanns-Clausen M, Kneisel S, Hutter M, Szabo B, Auwärter V (2013). Acute intoxication by synthetic cannabinoids—four case reports. Drug Test Anal.

[20] Hasegawa K, Minakata K, Gonmori K, Nozawa H, Yamagishi I, Watanabe K, Suzuki O (2017). Identification and quantification of predominant metabolites of synthetic cannabinoid MAB-CHMINACA in an authentic human urine specimen. Drug Test Anal.

